# Benzyl *N*′-(4,6-dimeth­oxy-2-methyl-3-phenyl-1*H*-indol-7-ylmethyl­ene)hydrazinecarbodithio­ate

**DOI:** 10.1107/S1600536808038592

**Published:** 2008-11-26

**Authors:** Hamid Khaldei, Hapipah Mohd Ali, Seik Weng Ng

**Affiliations:** a Department of Chemistry, University of Malaya, 50603 Kuala Lumpur, Malaysia

## Abstract

The asymmetric unit of the title compound, C_26_H_25_N_3_O_2_S_2_, contains two independent mol­ecules, which are linked by a pair of N—H⋯S hydrogen bonds, forming a dimer.

## Related literature

For a list of references of the benzyl esters of hydrazinecarbodithioic acids, see: Khaledi *et al.* (2008 ▶). For further synthetic details, see: Ali & Tarafder (1977 ▶); Jones *et al.* (2005 ▶).
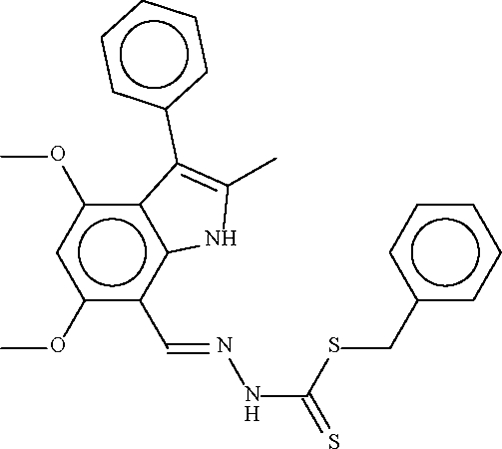


## Experimental

### 

#### Crystal data

C_26_H_25_N_3_O_2_S_2_
*M* *_r_* = 475.61
Triclinic, 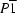

*a* = 10.0913 (2) Å
*b* = 12.8541 (3) Å
*c* = 18.9232 (4) Å
α = 90.461 (1)°
β = 103.960 (1)°
γ = 93.944 (1)°
*V* = 2375.71 (9) Å^3^
*Z* = 4
Mo *K*α radiation
μ = 0.25 mm^−1^
*T* = 100 (2) K
0.40 × 0.15 × 0.10 mm

#### Data collection

Bruker SMART APEX CCD diffractometer
Absorption correction: multi-scan (*SADABS*; Sheldrick, 1996 ▶) *T*_min_ = 0.906, *T*_max_ = 0.975
22819 measured reflections
10887 independent reflections
7429 reflections with *I* > 2σ(*I*)
*R*_int_ = 0.037

#### Refinement

*R*[*F* ^2^ > 2σ(*F* ^2^)] = 0.048
*wR*(*F* ^2^) = 0.145
*S* = 1.01
10887 reflections
601 parameters
H-atom parameters constrained
Δρ_max_ = 0.38 e Å^−3^
Δρ_min_ = −0.33 e Å^−3^

### 

Data collection: *APEX2* (Bruker, 2007 ▶); cell refinement: *SAINT* (Bruker, 2007 ▶); data reduction: *SAINT*; program(s) used to solve structure: *SHELXS97* (Sheldrick, 2008 ▶); program(s) used to refine structure: *SHELXL97* (Sheldrick, 2008 ▶); molecular graphics: *X-SEED* (Barbour, 2001 ▶); software used to prepare material for publication: *pubCIF* (Westrip, 2008 ▶).

## Supplementary Material

Crystal structure: contains datablocks global, I. DOI: 10.1107/S1600536808038592/hb2850sup1.cif

Structure factors: contains datablocks I. DOI: 10.1107/S1600536808038592/hb2850Isup2.hkl

Additional supplementary materials:  crystallographic information; 3D view; checkCIF report

## Figures and Tables

**Table 1:** Hydrogen-bond geometry (Å, °)

*D*—H⋯*A*	*D*—H	H⋯*A*	*D*⋯*A*	*D*—H⋯*A*
N1—H1⋯S4	0.88	2.47	3.330 (2)	164
N4—H4⋯S2	0.88	2.50	3.368 (2)	170

**Fig. 1.**
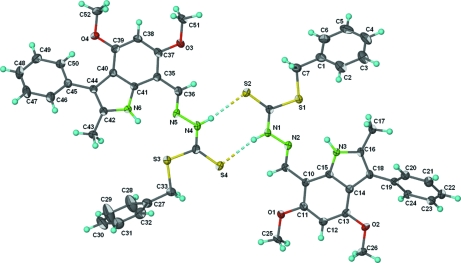
The molecular structure of (I) at the 70% probability level. Hydrogen atoms are drawn as spheres of arbitrary radius. Dashed lines denote hydrogen bonds.

## supplementary crystallographic information

### Comment

For a list of references of the benzyl esters of hydrazinecarbodithioic acids, see: Khaledi *et al.* (2008).  For further synthetic details, see: Ali & Tarafder (1977); Jones *et al.* (2005).

### Experimental

4,6-Dimethoxy-2-methyl-3-phenylindole-7-carbaldehyde was prepared by using a literature method (Jones *et al.*, 2005) as was S-benzyl dithiocarbazate (Ali & Tarafder, 1977). 4,6-Dimethoxy-2-methyl-3-phenylindole-7-carbaldehyde (0.59 g, 2 mmol) and *S*-benzyl dithiocarbazate (0.40 g, 2 mmol) were refluxed in ethanol (40 ml) for 6 h. About 1 ml acetic acid was also added. The precipitate was filtered, washed with cold ethanol and dried. Yellow prisms of (I) were grown by slow evaporation of a DMF solution at room temperature

### Refinement

Hydrogen atoms were placed at calculated positions (C–H = 0.95–0.99, N–H = 0.88Å) and refined as riding with *U*(H) = 1.2–1.5 times *U*_eq_(C,*N*).

### Crystal data

**Table:**

C_26_H_25_N_3_O_2_S_2_	*Z* = 4
*M**_r_* = 475.61	*F*_000_ = 1000
Triclinic, *P*1	*D*_x_ = 1.330 Mg m^−^^3^
Hall symbol: -P 1	Mo *K*α radiation λ = 0.71073 Å
*a* = 10.0913 (2) Å	Cell parameters from 3627 reflections
*b* = 12.8541 (3) Å	θ = 2.2–27.9º
*c* = 18.9232 (4) Å	µ = 0.25 mm^−^^1^
α = 90.461 (1)º	*T* = 100 (2) K
β = 103.960 (1)º	Prism, yellow
γ = 93.944 (1)º	0.40 × 0.15 × 0.10 mm
*V* = 2375.71 (9) Å^3^	

### Data collection

**Table:**

Bruker SMART APEX CCD diffractometer	10887 independent reflections
Radiation source: fine-focus sealed tube	7429 reflections with *I* > 2σ(*I*)
Monochromator: graphite	*R*_int_ = 0.037
*T* = 100(2) K	θ_max_ = 27.5º
ω scans	θ_min_ = 1.1º
Absorption correction: Multi-scan(SADABS; Sheldrick, 1996)	*h* = −13→13
*T*_min_ = 0.906, *T*_max_ = 0.975	*k* = −16→16
22819 measured reflections	*l* = −24→23

### Refinement

**Table:**

Refinement on *F*^2^	Secondary atom site location: difference Fourier map
Least-squares matrix: full	Hydrogen site location: inferred from neighbouring sites
*R*[*F*^2^ > 2σ(*F*^2^)] = 0.048	H-atom parameters constrained
*wR*(*F*^2^) = 0.146	*w* = 1/[σ^2^(*F*_o_^2^) + (0.0761*P*)^2^] where *P* = (*F*_o_^2^ + 2*F*_c_^2^)/3
*S* = 1.01	(Δ/σ)_max_ = 0.001
10887 reflections	Δρ_max_ = 0.38 e Å^−^^3^
601 parameters	Δρ_min_ = −0.33 e Å^−^^3^
Primary atom site location: structure-invariant direct methods	Extinction correction: none

### Special details

**Table:**

Geometry. All esds (except the esd in the dihedral angle between two l.s. planes) are estimated using the full covariance matrix. The cell esds are taken into account individually in the estimation of esds in distances, angles and torsion angles; correlations between esds in cell parameters are only used when they are defined by crystal symmetry. An approximate (isotropic) treatment of cell esds is used for estimating esds involving l.s. planes.

### Fractional atomic coordinates and isotropic or equivalent isotropic displacement parameters (Å^2^)

**Table:**

	*x*	*y*	*z*	*U*_iso_*/*U*_eq_	
S1	0.70730 (6)	0.77337 (4)	0.38863 (3)	0.02192 (14)	
S2	0.86760 (6)	0.64215 (4)	0.50774 (3)	0.02258 (14)	
S3	0.76473 (6)	0.21326 (4)	0.59896 (3)	0.02381 (15)	
S4	0.61229 (6)	0.34616 (5)	0.47915 (3)	0.02475 (15)	
N1	0.62636 (18)	0.58880 (14)	0.41970 (10)	0.0203 (4)	
H1	0.6232	0.5309	0.4439	0.024*	
N2	0.52245 (19)	0.60752 (14)	0.35981 (10)	0.0193 (4)	
N3	0.41658 (18)	0.70701 (13)	0.23268 (9)	0.0168 (4)	
H3	0.4872	0.7206	0.2700	0.020*	
N4	0.84585 (19)	0.40040 (14)	0.57324 (10)	0.0214 (4)	
H4	0.8499	0.4595	0.5504	0.026*	
N5	0.94526 (19)	0.38083 (14)	0.63461 (9)	0.0199 (4)	
N6	1.04365 (18)	0.27207 (13)	0.75800 (9)	0.0173 (4)	
H6	0.9726	0.2628	0.7205	0.021*	
O1	0.18934 (16)	0.40290 (12)	0.31166 (8)	0.0241 (4)	
O2	0.01662 (15)	0.56662 (12)	0.08209 (8)	0.0211 (3)	
O3	1.27723 (16)	0.58543 (12)	0.69482 (8)	0.0249 (4)	
O4	1.44777 (15)	0.39642 (12)	0.91349 (8)	0.0204 (3)	
C1	0.8502 (2)	0.95402 (18)	0.38428 (13)	0.0234 (5)	
C2	0.8241 (3)	0.95499 (19)	0.30847 (14)	0.0301 (6)	
H2	0.8166	0.8911	0.2817	0.036*	
C3	0.8088 (3)	1.0478 (2)	0.27173 (15)	0.0346 (6)	
H3A	0.7898	1.0476	0.2201	0.042*	
C4	0.8215 (3)	1.1411 (2)	0.31081 (16)	0.0365 (7)	
H4A	0.8129	1.2050	0.2858	0.044*	
C5	0.8463 (3)	1.1416 (2)	0.38548 (16)	0.0362 (7)	
H5	0.8540	1.2057	0.4120	0.043*	
C6	0.8600 (2)	1.04831 (19)	0.42195 (14)	0.0301 (6)	
H6A	0.8764	1.0490	0.4735	0.036*	
C7	0.8656 (2)	0.85233 (17)	0.42390 (13)	0.0240 (5)	
H7A	0.8807	0.8648	0.4770	0.029*	
H7B	0.9442	0.8171	0.4146	0.029*	
C8	0.7315 (2)	0.66011 (17)	0.44022 (11)	0.0181 (5)	
C9	0.4171 (2)	0.54120 (17)	0.34579 (12)	0.0192 (5)	
H9	0.4101	0.4862	0.3782	0.023*	
C10	0.3103 (2)	0.55083 (17)	0.28101 (11)	0.0170 (4)	
C11	0.1969 (2)	0.47839 (17)	0.26171 (12)	0.0191 (5)	
C12	0.0981 (2)	0.48203 (17)	0.19613 (11)	0.0187 (5)	
H12	0.0228	0.4311	0.1852	0.022*	
C13	0.1091 (2)	0.55997 (17)	0.14656 (11)	0.0176 (5)	
C14	0.2202 (2)	0.63542 (16)	0.16222 (11)	0.0165 (4)	
C15	0.3166 (2)	0.62922 (16)	0.22955 (11)	0.0163 (4)	
C16	0.3911 (2)	0.76147 (16)	0.16888 (11)	0.0166 (4)	
C17	0.4871 (2)	0.85195 (17)	0.16129 (12)	0.0218 (5)	
H17A	0.4500	0.8876	0.1160	0.033*	
H17B	0.5762	0.8270	0.1602	0.033*	
H17C	0.4983	0.9007	0.2028	0.033*	
C18	0.2714 (2)	0.72033 (16)	0.12301 (11)	0.0173 (4)	
C19	0.2177 (2)	0.75254 (17)	0.04715 (11)	0.0189 (5)	
C20	0.3054 (2)	0.76263 (17)	0.00017 (12)	0.0228 (5)	
H20	0.3986	0.7483	0.0172	0.027*	
C21	0.2575 (2)	0.79344 (19)	−0.07118 (12)	0.0271 (5)	
H21	0.3179	0.7993	−0.1027	0.033*	
C22	0.1218 (3)	0.81568 (19)	−0.09653 (13)	0.0285 (6)	
H22	0.0892	0.8376	−0.1451	0.034*	
C23	0.0345 (2)	0.80567 (18)	−0.05034 (12)	0.0252 (5)	
H23	−0.0585	0.8207	−0.0674	0.030*	
C24	0.0814 (2)	0.77397 (17)	0.02037 (12)	0.0203 (5)	
H24	0.0198	0.7667	0.0512	0.024*	
C25	0.0776 (2)	0.32470 (17)	0.29397 (13)	0.0256 (5)	
H25A	0.0825	0.2777	0.3349	0.038*	
H25B	0.0830	0.2847	0.2506	0.038*	
H25C	−0.0091	0.3582	0.2843	0.038*	
C26	−0.0941 (2)	0.48780 (17)	0.06387 (12)	0.0214 (5)	
H26A	−0.1508	0.5000	0.0152	0.032*	
H26B	−0.1499	0.4903	0.0996	0.032*	
H26C	−0.0575	0.4191	0.0643	0.032*	
C27	0.6207 (2)	0.03069 (18)	0.59376 (13)	0.0245 (5)	
C28	0.5762 (3)	0.0196 (2)	0.65644 (16)	0.0462 (8)	
H28	0.5381	0.0764	0.6750	0.056*	
C29	0.5862 (4)	−0.0736 (2)	0.69306 (18)	0.0581 (10)	
H29	0.5548	−0.0801	0.7364	0.070*	
C30	0.6409 (3)	−0.1562 (2)	0.66741 (16)	0.0391 (7)	
H30	0.6501	−0.2191	0.6936	0.047*	
C31	0.6823 (3)	−0.1476 (2)	0.60373 (15)	0.0362 (6)	
H31	0.7176	−0.2054	0.5847	0.043*	
C32	0.6726 (3)	−0.0545 (2)	0.56714 (14)	0.0310 (6)	
H32	0.7019	−0.0489	0.5232	0.037*	
C33	0.6132 (2)	0.13207 (18)	0.55449 (13)	0.0240 (5)	
H33A	0.5295	0.1660	0.5576	0.029*	
H33B	0.6112	0.1200	0.5025	0.029*	
C34	0.7437 (2)	0.32828 (17)	0.54922 (12)	0.0202 (5)	
C35	1.1528 (2)	0.43417 (16)	0.71813 (11)	0.0179 (5)	
C36	1.0496 (2)	0.44792 (17)	0.65222 (11)	0.0193 (5)	
H36	1.0583	0.5056	0.6221	0.023*	
C37	1.2684 (2)	0.50485 (17)	0.74051 (12)	0.0200 (5)	
C38	1.3671 (2)	0.49319 (17)	0.80491 (12)	0.0191 (5)	
H38	1.4446	0.5420	0.8179	0.023*	
C39	1.3533 (2)	0.41075 (17)	0.85036 (11)	0.0176 (4)	
C40	1.2386 (2)	0.33879 (16)	0.83225 (11)	0.0161 (4)	
C41	1.1435 (2)	0.35199 (16)	0.76586 (11)	0.0160 (4)	
C42	1.0701 (2)	0.20814 (16)	0.81719 (12)	0.0178 (5)	
C43	0.9715 (2)	0.11756 (17)	0.82004 (12)	0.0229 (5)	
H43A	0.9975	0.0852	0.8677	0.034*	
H43B	0.9731	0.0664	0.7816	0.034*	
H43C	0.8791	0.1414	0.8127	0.034*	
C44	1.1897 (2)	0.24588 (16)	0.86497 (11)	0.0166 (4)	
C45	1.2495 (2)	0.20264 (17)	0.93687 (12)	0.0195 (5)	
C46	1.2650 (2)	0.09692 (18)	0.94606 (13)	0.0270 (5)	
H46	1.2390	0.0507	0.9048	0.032*	
C47	1.3176 (3)	0.0574 (2)	1.01393 (14)	0.0383 (7)	
H47	1.3266	−0.0155	1.0188	0.046*	
C48	1.3569 (3)	0.1223 (2)	1.07449 (14)	0.0367 (7)	
H48	1.3931	0.0949	1.1211	0.044*	
C49	1.3433 (2)	0.2282 (2)	1.06674 (13)	0.0279 (6)	
H49	1.3708	0.2740	1.1082	0.033*	
C50	1.2897 (2)	0.26779 (18)	0.99898 (12)	0.0219 (5)	
H50	1.2800	0.3406	0.9945	0.026*	
C51	1.3965 (2)	0.65652 (17)	0.71292 (13)	0.0248 (5)	
H51A	1.3928	0.7072	0.6742	0.037*	
H51B	1.4785	0.6178	0.7180	0.037*	
H51C	1.3999	0.6933	0.7590	0.037*	
C52	1.5632 (2)	0.47112 (18)	0.93250 (13)	0.0233 (5)	
H52A	1.6237	0.4522	0.9786	0.035*	
H52B	1.5316	0.5405	0.9378	0.035*	
H52C	1.6132	0.4718	0.8941	0.035*	

### Atomic displacement parameters (Å^2^)

**Table:**

	*U*^11^	*U*^22^	*U*^33^	*U*^12^	*U*^13^	*U*^23^
S1	0.0212 (3)	0.0182 (3)	0.0231 (3)	−0.0008 (2)	−0.0005 (2)	0.0060 (2)
S2	0.0217 (3)	0.0223 (3)	0.0195 (3)	−0.0002 (2)	−0.0030 (2)	0.0042 (2)
S3	0.0245 (3)	0.0190 (3)	0.0235 (3)	−0.0007 (2)	−0.0023 (2)	0.0060 (2)
S4	0.0255 (3)	0.0232 (3)	0.0202 (3)	−0.0007 (2)	−0.0042 (2)	0.0051 (2)
N1	0.0210 (10)	0.0183 (10)	0.0185 (10)	−0.0001 (8)	−0.0009 (8)	0.0070 (8)
N2	0.0209 (10)	0.0190 (10)	0.0161 (9)	0.0012 (8)	0.0004 (7)	0.0031 (7)
N3	0.0159 (9)	0.0170 (9)	0.0156 (9)	−0.0008 (7)	0.0006 (7)	0.0035 (7)
N4	0.0241 (10)	0.0185 (10)	0.0193 (10)	0.0005 (8)	0.0011 (8)	0.0067 (8)
N5	0.0210 (10)	0.0213 (10)	0.0142 (9)	0.0016 (8)	−0.0019 (7)	0.0035 (8)
N6	0.0179 (9)	0.0152 (9)	0.0164 (9)	−0.0003 (7)	−0.0001 (7)	0.0008 (7)
O1	0.0255 (9)	0.0217 (8)	0.0206 (8)	−0.0073 (7)	−0.0008 (7)	0.0080 (7)
O2	0.0172 (8)	0.0232 (8)	0.0181 (8)	−0.0041 (6)	−0.0038 (6)	0.0021 (6)
O3	0.0262 (9)	0.0205 (8)	0.0244 (9)	−0.0060 (7)	0.0007 (7)	0.0077 (7)
O4	0.0156 (8)	0.0208 (8)	0.0214 (8)	−0.0027 (6)	−0.0011 (6)	0.0017 (6)
C1	0.0164 (11)	0.0212 (12)	0.0325 (13)	0.0000 (9)	0.0059 (10)	0.0007 (10)
C2	0.0337 (14)	0.0232 (13)	0.0379 (15)	0.0024 (11)	0.0172 (12)	0.0036 (11)
C3	0.0397 (16)	0.0309 (15)	0.0378 (15)	0.0023 (12)	0.0180 (12)	0.0100 (12)
C4	0.0293 (14)	0.0256 (14)	0.0568 (19)	0.0000 (11)	0.0149 (13)	0.0158 (13)
C5	0.0280 (14)	0.0189 (13)	0.0583 (19)	−0.0038 (11)	0.0059 (13)	−0.0034 (12)
C6	0.0244 (13)	0.0258 (13)	0.0362 (15)	−0.0052 (10)	0.0021 (11)	−0.0013 (11)
C7	0.0190 (12)	0.0205 (12)	0.0299 (13)	−0.0001 (9)	0.0011 (10)	0.0026 (10)
C8	0.0200 (11)	0.0173 (11)	0.0169 (11)	0.0009 (9)	0.0041 (9)	0.0022 (9)
C9	0.0221 (11)	0.0166 (11)	0.0181 (11)	−0.0006 (9)	0.0038 (9)	0.0037 (9)
C10	0.0181 (11)	0.0183 (11)	0.0140 (10)	0.0009 (9)	0.0030 (8)	0.0010 (8)
C11	0.0193 (11)	0.0175 (11)	0.0205 (11)	−0.0002 (9)	0.0050 (9)	0.0044 (9)
C12	0.0182 (11)	0.0196 (11)	0.0171 (11)	−0.0020 (9)	0.0027 (9)	0.0001 (9)
C13	0.0170 (11)	0.0207 (11)	0.0145 (11)	0.0030 (9)	0.0021 (8)	−0.0010 (9)
C14	0.0160 (11)	0.0175 (11)	0.0160 (11)	0.0024 (9)	0.0034 (8)	0.0003 (8)
C15	0.0172 (11)	0.0152 (11)	0.0162 (11)	−0.0005 (8)	0.0041 (8)	0.0006 (8)
C16	0.0170 (11)	0.0170 (11)	0.0162 (11)	0.0035 (9)	0.0045 (8)	0.0047 (8)
C17	0.0240 (12)	0.0210 (12)	0.0185 (11)	−0.0012 (9)	0.0021 (9)	0.0052 (9)
C18	0.0190 (11)	0.0159 (11)	0.0169 (11)	0.0028 (9)	0.0036 (8)	0.0035 (8)
C19	0.0208 (11)	0.0173 (11)	0.0165 (11)	−0.0029 (9)	0.0019 (9)	0.0020 (9)
C20	0.0217 (12)	0.0230 (12)	0.0220 (12)	−0.0010 (9)	0.0025 (9)	0.0032 (10)
C21	0.0299 (13)	0.0311 (14)	0.0198 (12)	−0.0049 (11)	0.0067 (10)	0.0056 (10)
C22	0.0335 (14)	0.0278 (14)	0.0188 (12)	−0.0073 (11)	−0.0017 (10)	0.0078 (10)
C23	0.0245 (12)	0.0217 (12)	0.0248 (13)	−0.0013 (10)	−0.0026 (10)	0.0040 (10)
C24	0.0216 (12)	0.0188 (12)	0.0191 (11)	−0.0005 (9)	0.0028 (9)	0.0024 (9)
C25	0.0278 (13)	0.0189 (12)	0.0268 (13)	−0.0069 (10)	0.0022 (10)	0.0049 (10)
C26	0.0179 (11)	0.0219 (12)	0.0216 (12)	−0.0025 (9)	0.0005 (9)	−0.0005 (9)
C27	0.0241 (12)	0.0207 (12)	0.0272 (13)	−0.0010 (10)	0.0042 (10)	0.0003 (10)
C28	0.072 (2)	0.0321 (16)	0.0483 (18)	0.0202 (15)	0.0366 (16)	0.0111 (13)
C29	0.087 (3)	0.0443 (19)	0.061 (2)	0.0224 (18)	0.048 (2)	0.0269 (16)
C30	0.0377 (16)	0.0240 (14)	0.0577 (19)	0.0025 (12)	0.0152 (14)	0.0150 (13)
C31	0.0368 (15)	0.0245 (14)	0.0447 (17)	0.0063 (12)	0.0036 (13)	−0.0018 (12)
C32	0.0305 (14)	0.0316 (14)	0.0304 (14)	0.0048 (11)	0.0058 (11)	−0.0009 (11)
C33	0.0205 (12)	0.0227 (12)	0.0262 (13)	−0.0032 (9)	0.0018 (10)	0.0025 (10)
C34	0.0228 (12)	0.0206 (12)	0.0164 (11)	0.0023 (9)	0.0030 (9)	0.0026 (9)
C35	0.0198 (11)	0.0158 (11)	0.0175 (11)	0.0027 (9)	0.0027 (9)	0.0017 (9)
C36	0.0231 (12)	0.0171 (11)	0.0179 (11)	0.0029 (9)	0.0047 (9)	0.0030 (9)
C37	0.0226 (12)	0.0150 (11)	0.0237 (12)	0.0028 (9)	0.0079 (9)	0.0031 (9)
C38	0.0172 (11)	0.0162 (11)	0.0229 (12)	−0.0020 (9)	0.0042 (9)	0.0003 (9)
C39	0.0166 (11)	0.0179 (11)	0.0175 (11)	0.0031 (9)	0.0024 (8)	−0.0017 (9)
C40	0.0172 (11)	0.0151 (11)	0.0158 (11)	0.0024 (8)	0.0034 (8)	0.0005 (8)
C41	0.0169 (11)	0.0139 (10)	0.0166 (11)	0.0005 (8)	0.0031 (8)	0.0002 (8)
C42	0.0193 (11)	0.0158 (11)	0.0180 (11)	0.0011 (9)	0.0042 (9)	0.0012 (9)
C43	0.0248 (12)	0.0187 (12)	0.0222 (12)	−0.0038 (9)	0.0017 (9)	0.0005 (9)
C44	0.0186 (11)	0.0139 (10)	0.0172 (11)	0.0011 (8)	0.0041 (8)	0.0007 (8)
C45	0.0169 (11)	0.0212 (12)	0.0201 (11)	0.0000 (9)	0.0041 (9)	0.0050 (9)
C46	0.0335 (14)	0.0222 (13)	0.0228 (12)	0.0041 (11)	0.0014 (10)	0.0027 (10)
C47	0.0569 (18)	0.0223 (14)	0.0312 (15)	0.0051 (13)	0.0008 (13)	0.0099 (11)
C48	0.0413 (16)	0.0384 (16)	0.0261 (14)	0.0029 (13)	−0.0006 (12)	0.0151 (12)
C49	0.0273 (13)	0.0331 (14)	0.0195 (12)	−0.0048 (11)	0.0003 (10)	0.0010 (10)
C50	0.0206 (12)	0.0218 (12)	0.0220 (12)	−0.0015 (9)	0.0035 (9)	0.0007 (9)
C51	0.0277 (13)	0.0185 (12)	0.0278 (13)	−0.0036 (10)	0.0073 (10)	0.0043 (10)
C52	0.0181 (11)	0.0234 (12)	0.0260 (13)	−0.0036 (9)	0.0016 (9)	−0.0017 (10)

### Geometric parameters (Å, °)

**Table:**

S1—C8	1.758 (2)	C20—H20	0.9500
S1—C7	1.811 (2)	C21—C22	1.388 (3)
S2—C8	1.666 (2)	C21—H21	0.9500
S3—C34	1.757 (2)	C22—C23	1.384 (3)
S3—C33	1.812 (2)	C22—H22	0.9500
S4—C34	1.664 (2)	C23—C24	1.382 (3)
N1—C8	1.333 (3)	C23—H23	0.9500
N1—N2	1.381 (2)	C24—H24	0.9500
N1—H1	0.8800	C25—H25A	0.9800
N2—C9	1.290 (3)	C25—H25B	0.9800
N3—C15	1.361 (3)	C25—H25C	0.9800
N3—C16	1.379 (3)	C26—H26A	0.9800
N3—H3	0.8800	C26—H26B	0.9800
N4—C34	1.328 (3)	C26—H26C	0.9800
N4—N5	1.377 (2)	C27—C28	1.371 (3)
N4—H4	0.8800	C27—C32	1.387 (3)
N5—C36	1.290 (3)	C27—C33	1.503 (3)
N6—C41	1.370 (3)	C28—C29	1.386 (4)
N6—C42	1.379 (3)	C28—H28	0.9500
N6—H6	0.8800	C29—C30	1.367 (4)
O1—C11	1.371 (2)	C29—H29	0.9500
O1—C25	1.432 (3)	C30—C31	1.370 (4)
O2—C13	1.353 (2)	C30—H30	0.9500
O2—C26	1.431 (2)	C31—C32	1.384 (3)
O3—C37	1.367 (3)	C31—H31	0.9500
O3—C51	1.430 (3)	C32—H32	0.9500
O4—C39	1.360 (2)	C33—H33A	0.9900
O4—C52	1.430 (3)	C33—H33B	0.9900
C1—C6	1.386 (3)	C35—C37	1.405 (3)
C1—C2	1.395 (3)	C35—C41	1.408 (3)
C1—C7	1.511 (3)	C35—C36	1.440 (3)
C2—C3	1.385 (3)	C36—H36	0.9500
C2—H2	0.9500	C37—C38	1.392 (3)
C3—C4	1.385 (4)	C38—C39	1.391 (3)
C3—H3A	0.9500	C38—H38	0.9500
C4—C5	1.374 (4)	C39—C40	1.404 (3)
C4—H4A	0.9500	C40—C41	1.404 (3)
C5—C6	1.387 (3)	C40—C44	1.460 (3)
C5—H5	0.9500	C42—C44	1.377 (3)
C6—H6A	0.9500	C42—C43	1.489 (3)
C7—H7A	0.9900	C43—H43A	0.9800
C7—H7B	0.9900	C43—H43B	0.9800
C9—C10	1.436 (3)	C43—H43C	0.9800
C9—H9	0.9500	C44—C45	1.479 (3)
C10—C11	1.398 (3)	C45—C46	1.386 (3)
C10—C15	1.416 (3)	C45—C50	1.398 (3)
C11—C12	1.395 (3)	C46—C47	1.381 (3)
C12—C13	1.395 (3)	C46—H46	0.9500
C12—H12	0.9500	C47—C48	1.374 (4)
C13—C14	1.404 (3)	C47—H47	0.9500
C14—C15	1.410 (3)	C48—C49	1.383 (4)
C14—C18	1.459 (3)	C48—H48	0.9500
C16—C18	1.375 (3)	C49—C50	1.382 (3)
C16—C17	1.492 (3)	C49—H49	0.9500
C17—H17A	0.9800	C50—H50	0.9500
C17—H17B	0.9800	C51—H51A	0.9800
C17—H17C	0.9800	C51—H51B	0.9800
C18—C19	1.480 (3)	C51—H51C	0.9800
C19—C24	1.394 (3)	C52—H52A	0.9800
C19—C20	1.398 (3)	C52—H52B	0.9800
C20—C21	1.390 (3)	C52—H52C	0.9800
			
C8—S1—C7	102.87 (10)	H25A—C25—H25B	109.5
C34—S3—C33	102.23 (11)	O1—C25—H25C	109.5
C8—N1—N2	118.81 (17)	H25A—C25—H25C	109.5
C8—N1—H1	120.6	H25B—C25—H25C	109.5
N2—N1—H1	120.6	O2—C26—H26A	109.5
C9—N2—N1	116.74 (18)	O2—C26—H26B	109.5
C15—N3—C16	110.09 (17)	H26A—C26—H26B	109.5
C15—N3—H3	125.0	O2—C26—H26C	109.5
C16—N3—H3	125.0	H26A—C26—H26C	109.5
C34—N4—N5	118.62 (18)	H26B—C26—H26C	109.5
C34—N4—H4	120.7	C28—C27—C32	118.3 (2)
N5—N4—H4	120.7	C28—C27—C33	121.1 (2)
C36—N5—N4	116.84 (18)	C32—C27—C33	120.6 (2)
C41—N6—C42	110.47 (17)	C27—C28—C29	120.7 (3)
C41—N6—H6	124.8	C27—C28—H28	119.7
C42—N6—H6	124.8	C29—C28—H28	119.7
C11—O1—C25	117.96 (17)	C30—C29—C28	120.6 (3)
C13—O2—C26	117.78 (17)	C30—C29—H29	119.7
C37—O3—C51	117.87 (17)	C28—C29—H29	119.7
C39—O4—C52	117.16 (17)	C29—C30—C31	119.5 (3)
C6—C1—C2	118.3 (2)	C29—C30—H30	120.2
C6—C1—C7	121.1 (2)	C31—C30—H30	120.2
C2—C1—C7	120.5 (2)	C30—C31—C32	119.9 (2)
C3—C2—C1	120.9 (2)	C30—C31—H31	120.0
C3—C2—H2	119.6	C32—C31—H31	120.0
C1—C2—H2	119.6	C31—C32—C27	121.0 (2)
C2—C3—C4	119.6 (3)	C31—C32—H32	119.5
C2—C3—H3A	120.2	C27—C32—H32	119.5
C4—C3—H3A	120.2	C27—C33—S3	106.50 (15)
C5—C4—C3	120.4 (2)	C27—C33—H33A	110.4
C5—C4—H4A	119.8	S3—C33—H33A	110.4
C3—C4—H4A	119.8	C27—C33—H33B	110.4
C4—C5—C6	119.8 (2)	S3—C33—H33B	110.4
C4—C5—H5	120.1	H33A—C33—H33B	108.6
C6—C5—H5	120.1	N4—C34—S4	123.02 (17)
C1—C6—C5	121.0 (2)	N4—C34—S3	112.47 (16)
C1—C6—H6A	119.5	S4—C34—S3	124.51 (14)
C5—C6—H6A	119.5	C37—C35—C41	115.23 (19)
C1—C7—S1	106.11 (15)	C37—C35—C36	121.77 (19)
C1—C7—H7A	110.5	C41—C35—C36	123.0 (2)
S1—C7—H7A	110.5	N5—C36—C35	119.0 (2)
C1—C7—H7B	110.5	N5—C36—H36	120.5
S1—C7—H7B	110.5	C35—C36—H36	120.5
H7A—C7—H7B	108.7	O3—C37—C38	122.4 (2)
N1—C8—S2	122.94 (16)	O3—C37—C35	115.52 (19)
N1—C8—S1	112.08 (15)	C38—C37—C35	122.1 (2)
S2—C8—S1	124.97 (13)	C39—C38—C37	120.6 (2)
N2—C9—C10	119.78 (19)	C39—C38—H38	119.7
N2—C9—H9	120.1	C37—C38—H38	119.7
C10—C9—H9	120.1	O4—C39—C38	122.33 (19)
C11—C10—C15	115.09 (19)	O4—C39—C40	117.33 (18)
C11—C10—C9	121.99 (19)	C38—C39—C40	120.34 (19)
C15—C10—C9	122.76 (19)	C39—C40—C41	117.10 (19)
O1—C11—C12	121.99 (19)	C39—C40—C44	135.86 (19)
O1—C11—C10	115.42 (18)	C41—C40—C44	107.02 (18)
C12—C11—C10	122.6 (2)	N6—C41—C40	107.34 (18)
C13—C12—C11	120.5 (2)	N6—C41—C35	128.02 (19)
C13—C12—H12	119.8	C40—C41—C35	124.64 (19)
C11—C12—H12	119.8	C44—C42—N6	108.84 (19)
O2—C13—C12	122.81 (19)	C44—C42—C43	132.2 (2)
O2—C13—C14	117.04 (19)	N6—C42—C43	118.92 (18)
C12—C13—C14	120.16 (19)	C42—C43—H43A	109.5
C13—C14—C15	117.29 (19)	C42—C43—H43B	109.5
C13—C14—C18	135.7 (2)	H43A—C43—H43B	109.5
C15—C14—C18	106.84 (18)	C42—C43—H43C	109.5
N3—C15—C14	107.63 (18)	H43A—C43—H43C	109.5
N3—C15—C10	127.87 (19)	H43B—C43—H43C	109.5
C14—C15—C10	124.39 (19)	C42—C44—C40	106.34 (18)
C18—C16—N3	109.44 (18)	C42—C44—C45	125.6 (2)
C18—C16—C17	131.52 (19)	C40—C44—C45	127.95 (19)
N3—C16—C17	119.00 (18)	C46—C45—C50	117.4 (2)
C16—C17—H17A	109.5	C46—C45—C44	122.1 (2)
C16—C17—H17B	109.5	C50—C45—C44	120.6 (2)
H17A—C17—H17B	109.5	C47—C46—C45	121.3 (2)
C16—C17—H17C	109.5	C47—C46—H46	119.3
H17A—C17—H17C	109.5	C45—C46—H46	119.3
H17B—C17—H17C	109.5	C48—C47—C46	120.7 (2)
C16—C18—C14	105.94 (18)	C48—C47—H47	119.7
C16—C18—C19	124.81 (19)	C46—C47—H47	119.7
C14—C18—C19	129.0 (2)	C47—C48—C49	119.1 (2)
C24—C19—C20	118.1 (2)	C47—C48—H48	120.4
C24—C19—C18	122.2 (2)	C49—C48—H48	120.4
C20—C19—C18	119.7 (2)	C50—C49—C48	120.3 (2)
C21—C20—C19	120.7 (2)	C50—C49—H49	119.9
C21—C20—H20	119.6	C48—C49—H49	119.9
C19—C20—H20	119.6	C49—C50—C45	121.2 (2)
C22—C21—C20	120.2 (2)	C49—C50—H50	119.4
C22—C21—H21	119.9	C45—C50—H50	119.4
C20—C21—H21	119.9	O3—C51—H51A	109.5
C23—C22—C21	119.3 (2)	O3—C51—H51B	109.5
C23—C22—H22	120.3	H51A—C51—H51B	109.5
C21—C22—H22	120.3	O3—C51—H51C	109.5
C24—C23—C22	120.6 (2)	H51A—C51—H51C	109.5
C24—C23—H23	119.7	H51B—C51—H51C	109.5
C22—C23—H23	119.7	O4—C52—H52A	109.5
C23—C24—C19	121.0 (2)	O4—C52—H52B	109.5
C23—C24—H24	119.5	H52A—C52—H52B	109.5
C19—C24—H24	119.5	O4—C52—H52C	109.5
O1—C25—H25A	109.5	H52A—C52—H52C	109.5
O1—C25—H25B	109.5	H52B—C52—H52C	109.5
			
C8—N1—N2—C9	−173.9 (2)	C18—C19—C24—C23	178.9 (2)
C34—N4—N5—C36	173.4 (2)	C32—C27—C28—C29	1.6 (5)
C6—C1—C2—C3	0.3 (4)	C33—C27—C28—C29	−178.7 (3)
C7—C1—C2—C3	179.5 (2)	C27—C28—C29—C30	0.1 (5)
C1—C2—C3—C4	0.8 (4)	C28—C29—C30—C31	−2.0 (5)
C2—C3—C4—C5	−1.3 (4)	C29—C30—C31—C32	2.1 (4)
C3—C4—C5—C6	0.6 (4)	C30—C31—C32—C27	−0.3 (4)
C2—C1—C6—C5	−1.0 (4)	C28—C27—C32—C31	−1.5 (4)
C7—C1—C6—C5	179.8 (2)	C33—C27—C32—C31	178.8 (2)
C4—C5—C6—C1	0.5 (4)	C28—C27—C33—S3	83.8 (3)
C6—C1—C7—S1	120.9 (2)	C32—C27—C33—S3	−96.5 (2)
C2—C1—C7—S1	−58.3 (3)	C34—S3—C33—C27	−179.61 (16)
C8—S1—C7—C1	−177.53 (16)	N5—N4—C34—S4	175.72 (15)
N2—N1—C8—S2	−175.46 (15)	N5—N4—C34—S3	−4.9 (3)
N2—N1—C8—S1	5.2 (2)	C33—S3—C34—N4	179.96 (17)
C7—S1—C8—N1	−177.14 (16)	C33—S3—C34—S4	−0.65 (18)
C7—S1—C8—S2	3.54 (18)	N4—N5—C36—C35	176.60 (18)
N1—N2—C9—C10	−174.83 (18)	C37—C35—C36—N5	−179.6 (2)
N2—C9—C10—C11	177.1 (2)	C41—C35—C36—N5	−1.9 (3)
N2—C9—C10—C15	1.9 (3)	C51—O3—C37—C38	3.2 (3)
C25—O1—C11—C12	1.6 (3)	C51—O3—C37—C35	−176.68 (19)
C25—O1—C11—C10	−178.21 (19)	C41—C35—C37—O3	−179.32 (18)
C15—C10—C11—O1	−179.30 (18)	C36—C35—C37—O3	−1.4 (3)
C9—C10—C11—O1	5.1 (3)	C41—C35—C37—C38	0.8 (3)
C15—C10—C11—C12	0.8 (3)	C36—C35—C37—C38	178.7 (2)
C9—C10—C11—C12	−174.7 (2)	O3—C37—C38—C39	179.2 (2)
O1—C11—C12—C13	180.0 (2)	C35—C37—C38—C39	−0.9 (3)
C10—C11—C12—C13	−0.2 (3)	C52—O4—C39—C38	0.8 (3)
C26—O2—C13—C12	−2.0 (3)	C52—O4—C39—C40	−178.75 (18)
C26—O2—C13—C14	177.40 (18)	C37—C38—C39—O4	179.69 (19)
C11—C12—C13—O2	179.58 (19)	C37—C38—C39—C40	−0.7 (3)
C11—C12—C13—C14	0.2 (3)	O4—C39—C40—C41	−178.01 (18)
O2—C13—C14—C15	179.65 (18)	C38—C39—C40—C41	2.4 (3)
C12—C13—C14—C15	−0.9 (3)	O4—C39—C40—C44	−0.3 (4)
O2—C13—C14—C18	−5.7 (4)	C38—C39—C40—C44	−179.9 (2)
C12—C13—C14—C18	173.8 (2)	C42—N6—C41—C40	0.5 (2)
C16—N3—C15—C14	−1.9 (2)	C42—N6—C41—C35	−179.0 (2)
C16—N3—C15—C10	174.5 (2)	C39—C40—C41—N6	177.86 (18)
C13—C14—C15—N3	178.33 (18)	C44—C40—C41—N6	−0.5 (2)
C18—C14—C15—N3	2.2 (2)	C39—C40—C41—C35	−2.6 (3)
C13—C14—C15—C10	1.7 (3)	C44—C40—C41—C35	179.0 (2)
C18—C14—C15—C10	−174.39 (19)	C37—C35—C41—N6	−179.6 (2)
C11—C10—C15—N3	−177.6 (2)	C36—C35—C41—N6	2.6 (3)
C9—C10—C15—N3	−2.0 (3)	C37—C35—C41—C40	1.1 (3)
C11—C10—C15—C14	−1.7 (3)	C36—C35—C41—C40	−176.8 (2)
C9—C10—C15—C14	173.9 (2)	C41—N6—C42—C44	−0.3 (2)
C15—N3—C16—C18	0.9 (2)	C41—N6—C42—C43	178.61 (19)
C15—N3—C16—C17	178.78 (19)	N6—C42—C44—C40	0.0 (2)
N3—C16—C18—C14	0.5 (2)	C43—C42—C44—C40	−178.7 (2)
C17—C16—C18—C14	−177.0 (2)	N6—C42—C44—C45	176.58 (19)
N3—C16—C18—C19	−174.34 (19)	C43—C42—C44—C45	−2.1 (4)
C17—C16—C18—C19	8.1 (4)	C39—C40—C44—C42	−177.6 (2)
C13—C14—C18—C16	−176.7 (2)	C41—C40—C44—C42	0.3 (2)
C15—C14—C18—C16	−1.7 (2)	C39—C40—C44—C45	5.9 (4)
C13—C14—C18—C19	−2.1 (4)	C41—C40—C44—C45	−176.2 (2)
C15—C14—C18—C19	172.9 (2)	C42—C44—C45—C46	48.5 (3)
C16—C18—C19—C24	−133.1 (2)	C40—C44—C45—C46	−135.6 (2)
C14—C18—C19—C24	53.3 (3)	C42—C44—C45—C50	−129.9 (2)
C16—C18—C19—C20	46.6 (3)	C40—C44—C45—C50	46.0 (3)
C14—C18—C19—C20	−127.0 (2)	C50—C45—C46—C47	0.3 (4)
C24—C19—C20—C21	0.1 (3)	C44—C45—C46—C47	−178.2 (2)
C18—C19—C20—C21	−179.6 (2)	C45—C46—C47—C48	−0.4 (4)
C19—C20—C21—C22	0.7 (4)	C46—C47—C48—C49	0.0 (4)
C20—C21—C22—C23	−0.8 (4)	C47—C48—C49—C50	0.5 (4)
C21—C22—C23—C24	0.1 (4)	C48—C49—C50—C45	−0.6 (4)
C22—C23—C24—C19	0.8 (3)	C46—C45—C50—C49	0.3 (3)
C20—C19—C24—C23	−0.9 (3)	C44—C45—C50—C49	178.8 (2)

### Hydrogen-bond geometry (Å, °)

**Table:**

*D*—H···*A*	*D*—H	H···*A*	*D*···*A*	*D*—H···*A*
N1—H1···S4	0.88	2.47	3.330 (2)	164
N4—H4···S2	0.88	2.50	3.368 (2)	170

## References

[bb1] Ali, M. A. & Tarafder, M. T. H. (1977). *J. Inorg. Nucl. Chem.* **39**, 1785-1788.

[bb2] Barbour, L. J. (2001). *J. Supramol. Chem.* **1**, 189–191.

[bb3] Bruker (2007). *APEX2* and *SAINT* Bruker AXS Inc., Madison, Wisconsin, USA.

[bb4] Jones, A. W., Wahyuningsih, T. W., Pchalek, K., Kumar, N. & Black, D. S. C. (2005). *Tetrahedron*, **61** 10490–10500.

[bb5] Khaledi, H., Mohd Ali, H. & Ng, S. W. (2008). *Acta Cryst.* E**64**, o2430. 10.1107/S1600536808038579 PMC2960023 21581398

[bb6] Sheldrick, G. M. (1996). *SADABS* University of Göttingen, Germany.

[bb7] Sheldrick, G. M. (2008). *Acta Cryst.* A**64**, 112–122. 10.1107/S0108767307043930 18156677

[bb8] Westrip, S. P. (2008). *publCIF* In preparation.

